# Impact of automated methods for quantitative evaluation of immunostaining: Towards digital pathology

**DOI:** 10.3389/fonc.2022.931035

**Published:** 2022-10-11

**Authors:** Nicolas Elie, Florence Giffard, Cécile Blanc-Fournier, Pierre-Marie Morice, Pierre-Emmanuel Brachet, Soizic Dutoit, Benoît Plancoulaine, Laurent Poulain

**Affiliations:** ^1^ Normandie Univ, UNICAEN, Federative Structure 4207 ‘Normandie Oncologie’, PLATON Services Unit, Virtual’His platform, Caen, France; ^2^ Normandie Univ, UNICAEN, Federative Structure 4207 ‘Normandie Oncologie’, PLATON Services Unit, Caen, France; ^3^ Normandie Univ, UNICAEN, Inserm U1086 ANTICIPE, Interdisciplinary Research Unit for Cancer Prevention and Treatment, Federative Structure 4207 ‘Normandie Oncologie’, F. Baclesse Comprehensive Cancer Centre, Caen, France; ^4^ UNICANCER, F. Baclesse Comprehensive Cancer Centre, Caen, France; ^5^ UNICANCER, F. Baclesse Comprehensive Cancer Centre, Biopathology Department, Caen, France; ^6^ Department of Pathology, Forensic Medicine and Pharmacology, Institute of Biomedical Sciences of the Faculty of Medicine, Vilnius University, Vilnius, Lithuania

**Keywords:** image processing, whole slide image, stereology, quality control, immunostaining evaluation

## Abstract

**Introduction:**

We sought to develop a novel method for a fully automated, robust quantification of protein biomarker expression within the epithelial component of high-grade serous ovarian tumors (HGSOC). Rather than defining thresholds for a given biomarker, the objective of this study in a small cohort of patients was to develop a method applicable to the many clinical situations in which immunomarkers need to be quantified. We aimed to quantify biomarker expression by correlating it with the heterogeneity of staining, using a non-subjective choice of scoring thresholds based on classical mathematical approaches. This could lead to a universal method for quantifying other immunohistochemical markers to guide pathologists in therapeutic decision-making.

**Methods:**

We studied a cohort of 25 cases of HGSOC for which three biomarkers predictive of the response observed *ex vivo* to the BH3 mimetic molecule ABT-737 had been previously validated by a pathologist. We calibrated our algorithms using Stereology analyses performed by two experts to detect immunohistochemical staining and epithelial/stromal compartments. Immunostaining quantification within Stereology grids of hexagons was then performed for each histological slice. To define thresholds from the staining distribution histograms and to classify staining within each hexagon as low, medium, or high, we used the Gaussian Mixture Model (GMM).

**Results:**

Stereology analysis of this calibration process produced a good correlation between the experts for both epithelium and immunostaining detection. There was also a good correlation between the experts and image processing. Image processing clearly revealed the respective proportions of low, medium, and high areas in a single tumor and showed that this parameter of heterogeneity could be included in a composite score, thus decreasing the level of discrepancy. Therefore, agreement with the pathologist was increased by taking heterogeneity into account.

**Conclusion and discussion:**

This simple, robust, calibrated method using basic tools and known parameters can be used to quantify and characterize the expression of protein biomarkers within the different tumor compartments. It is based on known mathematical thresholds and takes the intratumoral heterogeneity of staining into account. Although some discrepancies need to be diminished, correlation with the pathologist’s classification was satisfactory. The method is replicable and can be used to analyze other biological and medical issues. This non-subjective technique for assessing protein biomarker expression uses a fully automated choice of thresholds (GMM) and defined composite scores that take the intra-tumor heterogeneity of immunostaining into account. It could help to avoid the misclassification of patients and its subsequent negative impact on therapeutic care.

## Introduction

Protein expression and localization and some post-translational modifications are crucial to many biological processes. As a result, dysregulation is frequently associated with pathological disorders. A current focus of biologists and pathologists is to assess protein expression as accurately as possible. Such studies frequently include the evaluation of the intensity of protein expression level, its subcellular localization, and heterogeneity within whole tissue sections. Appropriate staining methods are required to evaluate these parameters on histological sections. One of these methods is immunohistochemistry, which is widely used in experimental research and in routine clinical practice in pathology laboratories.

The assessment of the intensity of expression level has become a hot topic (1, 2). This intensity can be influenced by many factors as a result of immuno-histochemical labeling (3). However, if the same conditions are applied according to a well-defined protocol (fixation time, same staining conditions), it is possible to compare expression levels (4). Such quantitative evaluations are regularly used in clinical practice for biomarkers such as Her2/neu, estrogen receptor (ER), and progesterone receptor (PR) for which testing guidelines have been established (5, 6). However, an evaluation of the percentage of positive cells and/or of global protein expression level is sometimes unable to provide sufficient relevant information. Moreover, the subjective perception of pathologists may create a bias. This is exemplified by numerous works showing both intra- and inter-observer variability in results (7–9). These discrepancies arise partly from the subjectivity of the measurement but also from the distribution and heterogeneity of the intensity of markers in whole tissue sections. Indeed, it is sometimes difficult to evaluate the presence of different foci with different staining intensities on the same section. Nevertheless, in some cases such as Ki-67 proliferative marker quantification, the contribution of the association to this section of the value of the most represented or strongest focus has been considered pertinent for some predictive purposes (10).

Nowadays, thanks to technologies such as digital slide images and automated image analysis, it is possible first to quantify the expression level and second to account for these heterogeneous components by integrating quantitative parameters of heterogeneity (11). In the search for an innovative automated quantitative method, we investigated the possibility of automatically quantifying the expression of biomarkers previously reported as able to predict the response to a BH3-mimetic molecule (ABT-737) in ovarian tumor slices cultivated and exposed *ex vivo* to this drug (12). This attempted to automatically evaluate staining intensity in whole-slide images of tumor tissues, based on systematic subsampling in a hexagonal tiling array. The technique is based on the Stereology theory (13), which allows non-biased results to be estimated accurately by means of grids (crosses, squares, hexagons, etc.).

Three different immunomarkers were evaluated comparatively by a pathologist and image analysis. We used the proteins Mcl-1, Bim, and P-ERK, which were identified as predictive biomarkers in a previous study and exhibited various expression patterns and histological heterogeneity. First, we used Stereology laws by using a grid of crosses to obtain reference values in order to adjust the image processing (IP) and to estimate the protein expression level revealed by 3,3′-diaminobenzidine (DAB)-labeled intensity in the hexagonal tiling. Second, we applied two methods to assess positive and negative cases relative to the protein expressions: the Gaussian Mixture Model (GMM) (14) considering only labeling intensity and principal component analysis (PCA), which also takes heterogeneity into account. This allowed us to propose a scoring method using the PCA from the expression levels of Bim, Mcl-1, and P-ERK.

## Materials and methods

### Eligibility

For this study, we used the data previously obtained in patients diagnosed with advanced high-grade serous ovarian cancer (HGSOC) and no prior chemotherapy exposure. Tumor nodules from the peritoneal carcinomatosis were obtained during initial surgery and used for the “ABT/CARBO *ex vivo*” study. The protocol received all necessary institutional approval, and all patients provided written informed consent (NCT01440504).

### Immunohistochemical analysis

Automated immunohistochemistry using a DakoCytomation Autostainer was performed on 4-μm-thick paraffin sections. The mouse monoclonal antibody anti-Mcl-1 (Y37) was obtained from Abcam (Paris, France). The rabbit monoclonal antibodies anti-Bim (C34C5) and Phospho-p44/42 MAPK (Thr202/Tyr204) (D13.14.4E) corresponding to Phospho-Erk1/2 and noted P-ERK were obtained from Cell Signaling (Ozyme, Saint Quentin Yvelines, France).

Immunohistochemistry procedures were as previously described (12). Briefly, to unmask epitopes, deparaffinized slides were treated for 15 min by a high-temperature-heating antigen retrieval technique in EDTA buffer 0.5M pH 8 for Bim (EL, L, and S isoforms) and Mcl-1 antibodies and in citrate buffer 0.07M pH6 for P-ERK antibody. Sections were incubated for 1 h at room temperature with the primary antibodies. After washing, slides were incubated with the Perox Detect System (Novocastra, Leica Microsystems, Nanterre, France), according to the manufacturer’s instructions. Staining was performed with DAB chromogen, and sections were counterstained with hematoxylin QS (Vector Laboratories, Clinisciences, Nanterre, France). Stained slides were then digitized with a ScanScope CS slide scanner (Leica Biosystems, Nussloch, Germany).

### Immunostaining evaluation by the pathologist

Marker quantification or classification was assessed as follows by an independent certified pathologist. For Mcl-1 and Bim, the intensity of the staining was scored as high or low according to the degree of homogeneity of the staining pattern observed. Regarding P-ERK, the expression of the phosphorylated forms of ERK was strongly heterogeneous in the tumor nodules, contrary to the other proteins. We then assessed these parameters using both the percentage of marked cells and the staining intensity. Staining was considered as high only if the percentage of stained cells with high intensity was at least equal to 50% (12).

### Digital acquisition

Whole-slide images (WSIs) of histological sections were digitized with a 20× objective (0.5 µm/pixel) using the ScanScope CS slide scanner. Images were recorded as tiled pyramidal tiff images.

*Image processing: ROI definition*. For each image, a region of interest (ROI) was drawn using the ImageScope software (Leica Biosystems, Nussloch, Germany) to remove the artifacts and keep only tumoral areas on histological sections. For subsequent analysis, all processing operations were applied only in the regions of interest.

### Immunostaining detection

Immunostaining detection was first assessed by a color space change and second by a pixel classification (13): (i) the Otha color space is particularly suited to histochemistry staining because the second layer can adjust the blue and red colors (15), and (ii) the algorithm used to search for the maximum likelihood had separated a color histogram by several normal distributions, called the “Gaussian Mixture Model” (14). The algorithm was adjusted relative to the Gaussian function parameters using a Stereology procedure to calibrate it.

### Epithelium detection

Epithelium segmentation was performed relative to the “time–frequency” wavelet algorithm proposed by Denis Gabor (16). One of its well-known applications is Gaussian beam sampling in optics (17). Moreover, the wavelet theory and especially Gabor’s tight frame improved the mathematical concept (18). These two functionalities allowed us to create a processing algorithm of digital images modeled by the Fourier transform with a two-dimensional sliding window: (i) Fourier transform with a Gaussian window on the intensity image, (ii) low-pass filtering weighted by a cosine, and then (iii) inverse Fourier transform with the previous Gaussian window. The IP was completed (i) by implementing a segmentation by moments to obtain a binary image and (ii) by performing a morphological opening operation to eliminate the residual noise and the small objects in the binary image (19).

### Quality control

This was performed using a Stereology test grid of crosses. This method was used to superpose an ROI located randomly with a regular network of points (20). This method consisted in superimposing a stereological grid of crosses at random in the region of interest (20). The image readers had to put two types of mark under each cross, positive or negative, depending on the color or the staining intensity. In routine practice, this two-mark process allows an estimation of the surface ratio with an uncertainty computation (21). Here, the method was applied to adjust the IP parameters by best matching the positive and negative crosses positioned by the readers with the positive and negative surfaces detected by IP. Then, a quality factor was evaluated from the uncertainty. Two types of quality factors were computed for the different algorithm types. The first was computed by comparing the confidence intervals (95%) between the crosses marked by the experts and the virtual marked crosses drawn inside the surfaces detected by the IP. Thus, the calculation of the overlap of the confidence intervals gave the first quality factor. The second was computed by searching for the true and false positive crosses and the true and false negative crosses relative to the expert cross set and to the virtual cross set established by the IP. Thus, the average of the sensitivity and the specificity gave the second quality factor (22).

### Overlay epithelium/immunostaining

To evaluate the immunostaining rate only within the epithelium, the detection image “OTHA” was associated with the detection image of the epithelium “GABOR.” The resulting image contained only the colored pixels in the epithelial territories.

Transmittance was performed using a Stereology test grid of hexagons. ROIs were regularly subdivided by hexagons to take the heterogeneity of immunostaining within the epithelium into account, a technique described previously (11). For each hexagon, the transmittance was calculated as follows:


$$\text{Transmittance}=\frac{\Sigma \left(\text{Brown intensity pixel }-255\right)}{\left(\Sigma^{\;}\text{pixels in epithelium}\right)\times 255}$$

Therefore, the transmittance was between 0 and 1.

### Gaussian thresholding

Automatic thresholds were established by applying the algorithm of the GMM (14). Three Gaussian functions were sought directly in a histogram by the GMM algorithm. The representative curves gave intersection points defining possible thresholds. Two thresholds delimited three areas that we termed low, middle, and high, respectively.

### Principal Component Analysis score

Principal Component Analysis (PCA) (23) was used to establish a biological score: (i) the first three statistical moments were computed from the hexagonal tiling, case per case, (ii) the PCA algorithm was applied to the previous data set, and (iii) the principal component with the maximum variance was considered as the score. This score was normalized as a percentage before analysis.

## Results

### Calibration and validation of quantitative evaluation of immunostaining tool

Three images were used for each marker: 2441 points for Bim, 6467 for Mcl-1, and 4735 for P-ERK.

**Figure 1:**
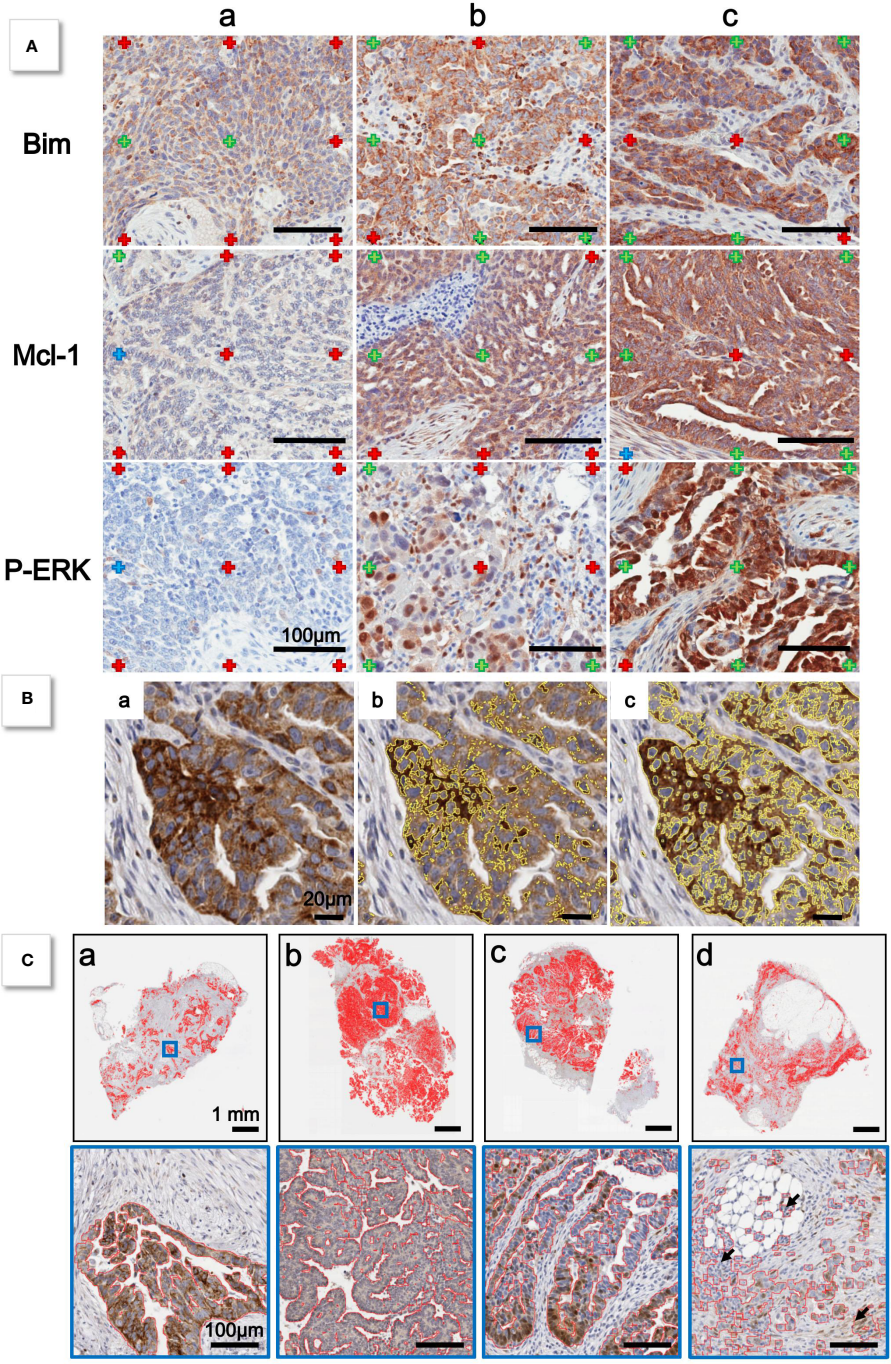
**(A)** Grid of cross is applied to histological sections. For each marker, a selection of sections is made to evaluate low **(a)**, medium **(b)**, and high **(c)** intensity staining. For each cross, expert indicates if labeled (green cross) or unlabeled (red cross) staining. **(B)** Detail of **(a)** histological section. Yellow lines indicate limits of detection of Otha method before calibration **(b)** and after calibration **(c)**. **(C)** Examples of epithelium detection after calibration by Gabor method (red lines).

Labeled and unlabeled marks were independently identified by the two experts on Stereology grids (**Figure 1A**). Two steps were needed to calibrate the labeled areas: (i) parameter adjustment of image processing (IP) and (ii) quality factor computation after calibration. IP calibration was based on the surface occupied by the labeled and unlabeled pixels. Grids with crosses were then used on three DAB-stained whole slide-images to detect Bim, Mcl-1, and P-ERK, respectively.

First, two experts examined the different grids and superimposed marks (labeled, unlabeled, and others). We kept only the marks common to the two experts in order to improve the IP setting. Then, calibration was checked simultaneously on three Bim cases, three Mcl-1 cases, and three P-ERK cases (**Table 1**). Second, the quality factor was established: first between the experts and then between the reference expert and IP performed after the calibration presented above (**Table 2**). **Figure 1B** shows that before the expert-based calibration, the staining detection performed by IP was unsatisfactory, whereas the calibration process allowed complete staining detection.

**Table 1:** Table of concordance of marks made on Stereology grids (one table/marker) by two experts (second column).

P15 Bim	Expert 1 vs. 2	Experts vs. IP	P25 Mcl-1	Expert 1 vs. 2	Experts vs. IP	P19 P-ERK	Expert 1 vs. 2	Experts vs. IP
Labeled mark	73.04%	99.33%	Labeled mark	86.53%	92.77%	Labeled mark	90.00%	89.08%
Unlabeled mark	96.17%	83.58%	Unlabeled mark	93.73%	91.87%	Unlabeled mark	98.32%	90.73%
Total mark	413	316	Total mark	1772	1483	Total mark	507	450
**P18 Bim**	**Expert 1 vs. 2**	**Experts vs. IP**	**P29 Mcl-1**	**Expert 1 vs. 2**	**Experts vs. IP**	P26 P-ERK	Expert 1 vs. 2	Experts vs. IP
Labeled mark	63.23%	96.94%	Labeled mark	71.96%	93.04%	Labeled mark	83.93%	100.00%
Unlabeled mark	87.31%	94.48%	Unlabeled mark	96.03%	50.46%	Unlabeled mark	99.06%	81.92%
Total mark	549	420	Total mark	1933	1066	Total mark	1541	1252
**P26 Bim**	**Expert 1 vs. 2**	**Experts vs. IP**	**P37 Mcl-1**	**Expert 1 vs. 2**	**Experts vs. IP**	P36 P-ERK	Expert 1 vs. 2	Experts vs. IP
Labeled mark	73.31%	99.51%	Labeled mark	83.78%	95.75%	Labeled mark	74.84%	89.08%
Unlabeled mark	82.39%	89.77%	Unlabeled mark	94.92%	84.05%	Unlabeled mark	97.89%	85.21%
Total mark	1479	1091	Total mark	2762	2207	Total mark	2687	2188

Marks common to both experts were used for calibration and then compared to IP (third column).

**Table 2:** Quality factors: factors between two experts and factor between reference expert and image processing (one table/marker).

	Expert 1					Expert 1				Expert 1		
	Sensitivity	Specificity	Quality Factor			Sensitivity	Specificity	Quality Factor		Sensitivity	Specificity	Quality Factor
**P15 Bim**			** **		**P25 Mcl-1**			**P19 P-ERK**		
**Expert 2**	70.90%	94.59%	82.75%	** ** ** **	**Expert 2**	70.90%	94.59%	82.75%	**Expert 2**	97.85%	92.05%	94.95%
**IP_OHTA**	54.44%	90.48%	72.46%	**IP_OHTA**	54.44%	90.48%	72.46%	**IP_OHTA**	89.43%	75.86%	82.65%
** **	**P18 Bim**			** **		**P29 Mcl-1**			** **	**P26 P-ERK**		
**Expert 2**	85.79%	66.22%	76.01%	** ** ** **	**Expert 2**	85.79%	66.22%	76.01%	**Expert 2**	99.39%	77.05%	88.22%
**IP_OHTA**	82.62%	62.28%	72.45%	**IP_OHTA**	82.62%	62.28%	72.45%	**IP_OHTA**	98.64%	45.71%	72.18%
** **	**P26 Bim**			** **		**P37 Mcl-1**			** **	**P36 P-ERK**		
**Expert 2**	92.94%	49.40%	71.17%	** ** ** **	**Expert 2**	92.94%	49.40%	71.17%	**Expert 2**	96.67%	82.64%	89.66%
**IP_OHTA**	89.82%	55.77%	72.80%	**IP_OHTA**	89.82%	55.77%	72.80%	**IP_OHTA**	91.52%	80.85%	86.19%

### Calibration and validation of quantitative evaluation of epithelium detection tools

As previously performed for immunostaining detection, three images were used for each marker to evaluate and calibrate the detection of epithelium. Then, the quality factor was established using inter-expert agreement. IP calibration was applied to the epithelium extraction to establish the best filter and most efficient size of the morphological opening. First, the intersection between the marked crosses of the experts and the IP masks allowed these two parameters to be adjusted. Then, the true-positive, true-negative, false-positive, and false-negative marks were found between the two experts. Finally, masks built by IP were compared to the marked crosses of the reference expert (**Table 3**).

**Table 3:** Quality factors between two experts (expert 1 defined as reference) and factor between reference expert and image processing were calculated from results obtained on four WSIs stained with DAB for each marker (one table/marker).

Expert 1				Expert 1						Expert 1						
Sensitivity	Specificity	Factor Quality		Sensitivity		Specificity		Factor Quality		Sensitivity		Specificity				Factor Quality
**P14 Bim**			**P14 Mcl-1**					**P14 P-ERK**						
100.00%	99.25%	99.63%	**Expert 2**	97.92%	99.06%		98.49%		**Expert 2**	97.44%	97.64%		97.54%			
76.67%	97.78%	87.23%	**IP_GABOR**	89.58%	92.45%		91.02%		**IP_GABOR**	61.54%	89.50%		75.52%			
**P24 Bim**			** **	**P24 Mcl-1**					** **	**P24 P-ERK**						
91.03%	98.47%	94.75%	**Expert 2**	94.24%	92.04%		93.14%		**Expert 2**	92.08%	95.62%		93.85%			
93.62%	89.47%	91.55%	**IP_GABOR**	86.84%	91.53%		89.19%		**IP_GABOR**	73.90%	90.14%		82.02%			
**P28 Bim**			** **	**P28 Mcl-1**					** **	**P28 P-ERK**						
93.89%	97.67%	95.78%	**Expert 2**	99.08%	93.00%		97.54%		**Expert 2**	98.23%	95.83%		97.03%			
95.20%	90.32%	92.76%	**IP_GABOR**	96.43%	80.20%		88.32%		**IP_GABOR**	86.84%	88.68%				87.76%	
**P32 Bim**			** **	**P32 Mcl-1**					** **	**P32 P-ERK**						
84.38%	94.64%	89.51%	**Expert 2**	93.42%	94.43%		93.93%		**Expert 2**	70.97%	98.43%			84.70%		
82.18%	74.62%	78.40%	**IP_GABOR**	88.46%	78.04%		83.25%		**IP_GABOR**	54.17%	74.62%			64.40%		

**Figure 1C** shows epithelium detection by IP on several cases.

### Application to automated immunostaining quantification

#### First approach: use of immunostaining classes

**Figure 2:**
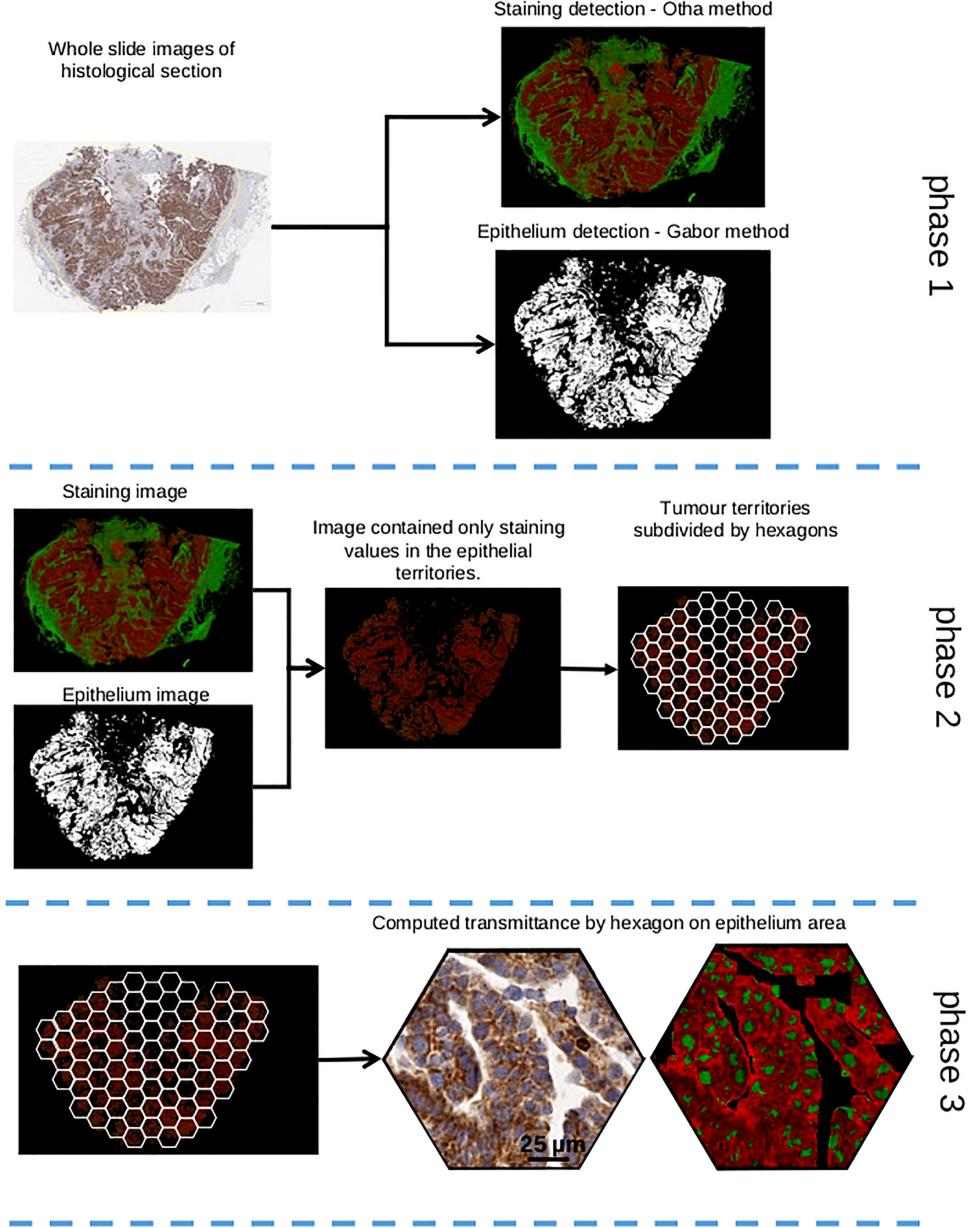
Image processing to evaluate staining of a marker. Phase 1: staining detection thanks to Otha method and epithelium detection with Gabor method. Phase 2: epithelium mask and staining image were mixed to keep only epithelium territories. This image is subdivided by hexagon pattern. Phase 3: in each hexagon, transmittance of staining is computed only in epithelial territories.

**Figure 3:**
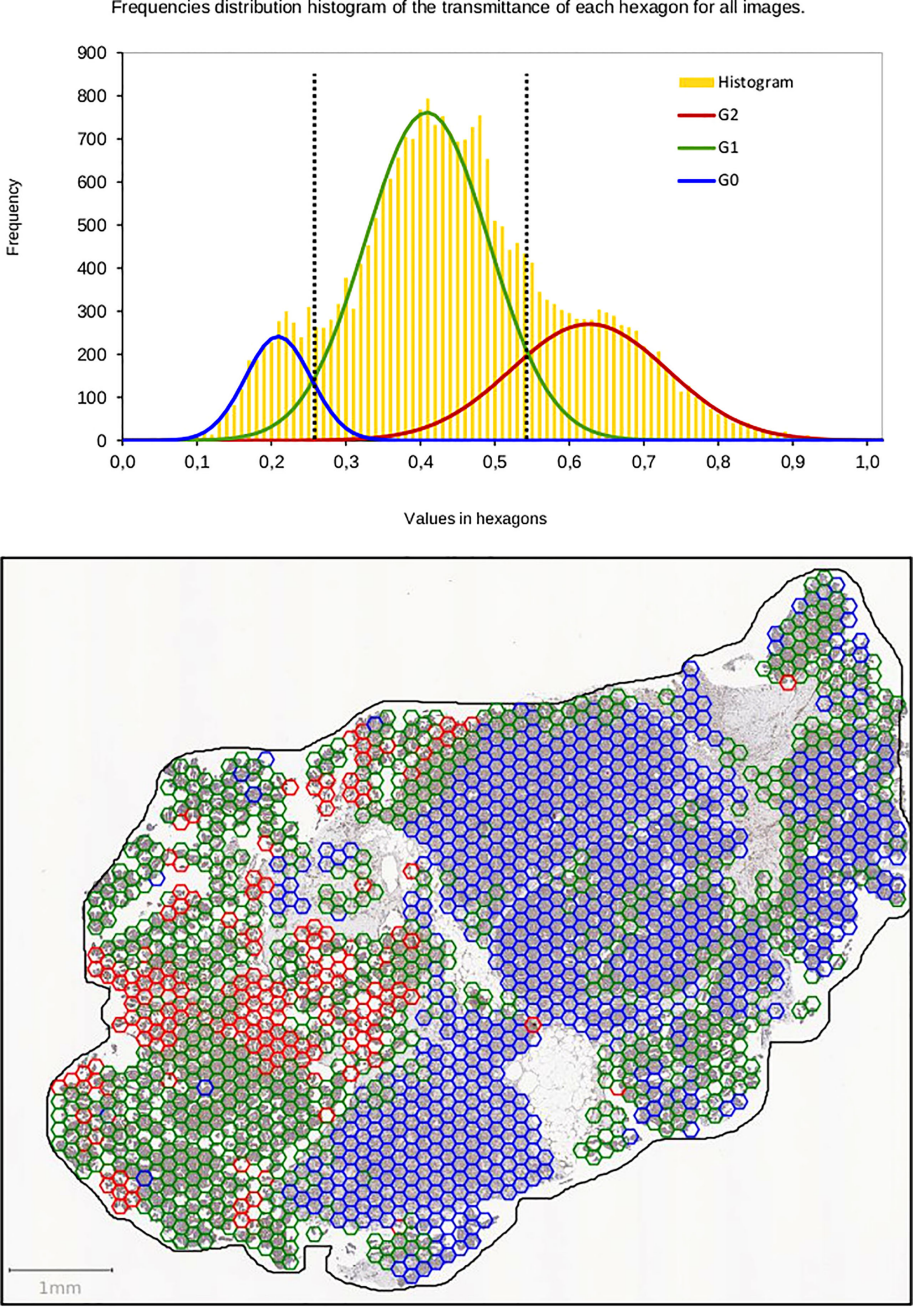
Automatic thresholds are determined by the algorithm of the Gaussian Mixture Model on distribution of frequencies of the transmittance for each marker. The threshold values correspond to crossing between Gaussians (dotted lines). The thresholds are used to classify hexagons as low, medium, and high intensity (hexagons blue, green, red).

**Figure 4:**
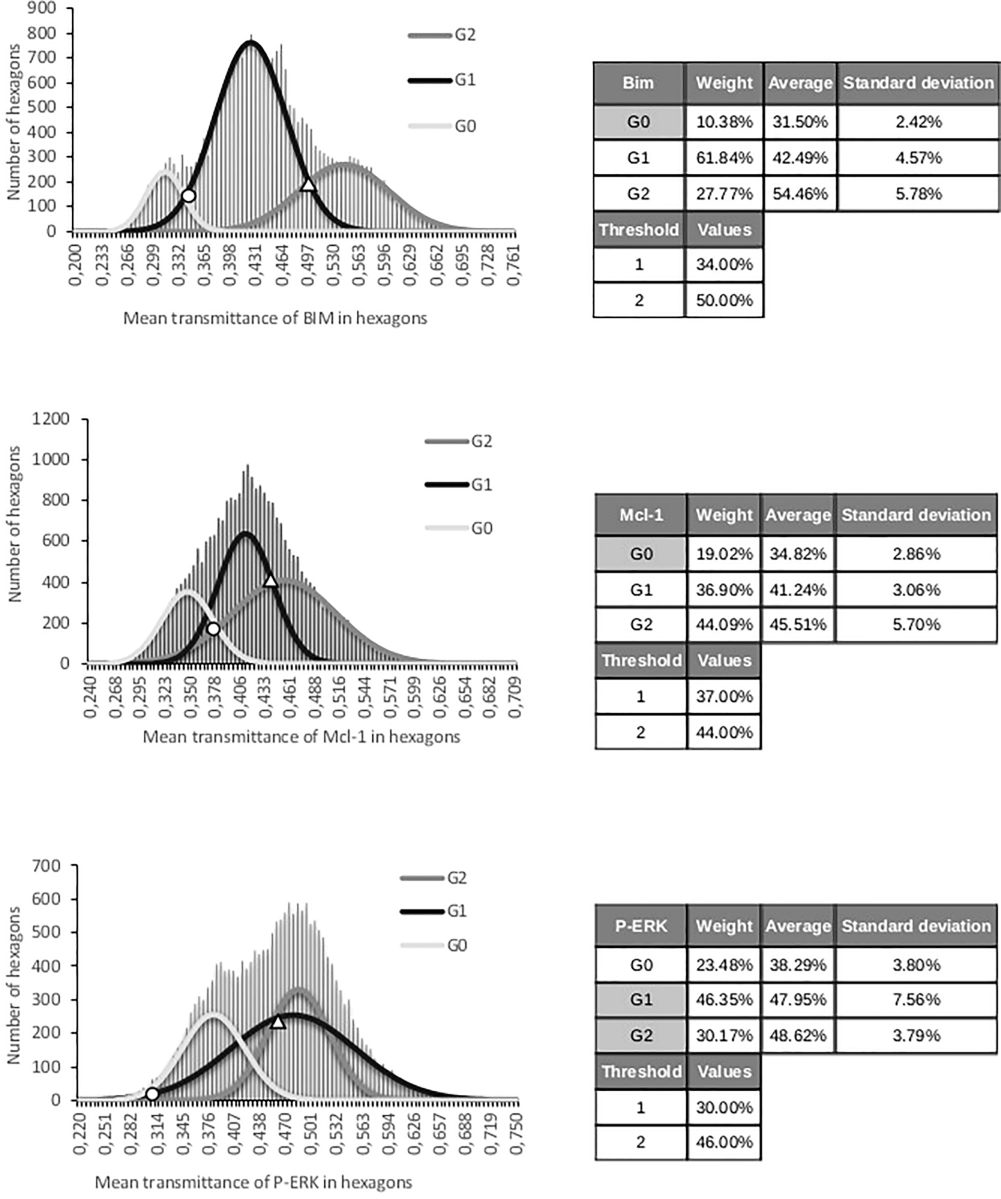
Distribution of mean transmittance of all hexagons for each marker (Bim, Mcl-1, and P-ERK). Parameters for three Gaussian functions and two thresholds for each set of hexagonal patterns (Bim, Mcl-1, and P-ERK). Circle and triangle indicate threshold values.

Next, we used the proposed method on all the WSI for each marker, as shown in **Figure 2**. Histograms were analyzed with the GMM. They were built by accumulating all hexagon values collected from the 25 WSIs for Bim, Mcl-1, and P-ERK independently. Two thresholds for each were found by intersecting the Gaussian curves (**Figure 3**). Thus, three classes were built for each IHC staining, termed entitled low, medium, and high (**Figure 4**).

The main class was defined as the one that contained the most hexagon patterns. The comparison with the estimation of the expert required grouping together two classes in order to keep only two classes called “low” and “high.” Thus, a given case could be considered “low” or “high” depending on the largest number of hexagons in classes 0, 1, or 2 obtained by IP (**Table 4**).

**Table 4:** Distribution in three classes by IP but only two by expert analysis.

Patient	Number of hexagons by class			Score IA Bim	Score Expert Bim	Patient	Number of hexagons by class			Score IA Mcl-1	Score Expert Mcl-1	Patient	Number of hexagons by class			R 50%	Score IA P-ERK	Score Expert P-ERK
	class 0	class 1	class 2				class 0	class 1	class 2				class 0	class 1	class 2			
P10	198	770	0	*high*	*high*	P10	919	36	2	**low**	**low**	P10	949	20	1	0.10%	low	low
P12	1204	162	0	low	low	**P12**	**653**	**246**	**37**	**low**	*high*	P12	589	514	20	1.78%	low	low
P13	512	415	0	low	low	P13	111	466	94	*high*	*high*	P13	278	322	438	42.20%	low	low
P14	0	366	143	*high*	*high*	P14	9	193	227	*high*	*high*	P14	210	420	23	3.52%	low	low
P15	0	92	338	*high*	*high*	P15	36	374	77	*high*	*high*	**P15**	**52**	**178**	**298**	**56.44%**	*high*	**low**
P17	0	8	2	*high*	*high*	**P17**	**71**	**53**	**54**	**low**	*high*	**P17**	**2**	**74**	**65**	**46.10%**	**low**	*high*
P18	5	649	9	*high*	*high*	P18	3	137	715	*high*	*high*	**P18**	**207**	**264**	**332**	**41.34%**	**low**	*high*
P19	0	42	297	*high*	*high*	P19	2	92	261	*high*	*high*	P19	44	52	343	78.13%	*high*	*high*
P21	10	1410	38	*high*	*high*	**P21**	**157**	**1005**	**209**	*high*	**low**	P21	1284	40	59	4.27%	low	low
P22	0	49	77	*high*	*high*	P22	0	61	146	*high*	*high*	**P22**	**0**	**30**	**0**	**0.00%**	**low**	*high*
P24	0	795	1367	*high*	*high*	**P24**	**784**	**807**	**181**	*high*	**low**	P24	896	302	745	38.34%	low	low
P25	2	707	942	*high*	*high*	P25	8	253	1664	*high*	*high*	P25	1225	389	420	20.65%	low	low
**P26**	**0**	**1791**	**58**	*high*	**low**	**P26**	**3**	**1007**	**1202**	*high*	**low**	P26	1112	529	10	0.61%	low	low
P27	27	849	175	*high*	*high*	P27	10	513	391	*high*	*high*	P27	593	664	88	6.54%	low	low
P28	0	60	768	*high*	*high*	P28	0	9	801	*high*	*high*	**P28**	**103**	**107**	**729**	**77.64%**	*high*	**low**
**P29**	**4**	**2129**	**114**	*high*	**low**	P29	1231	634	62	**low**	**low**	P29	577	1250	60	3.18%	low	low
P30	0	30	315	*high*	*high*	P30	21	291	136	*high*	*high*	**P30**	**57**	**138**	**253**	**56.47%**	*high*	**low**
P31	22	2107	240	*high*	*high*	**P31**	**410**	**1735**	**199**	*high*	**low**	P31	275	716	889	47.29%	low	low
**P32**	**711**	**636**	**4**	**low**	*high*	P32	654	301	22	*low*	*low*	P32	263	910	28	2.33%	low	low
P33	0	976	703	*high*	*high*	**P33**	**295**	**595**	**239**	*high*	**low**	**P33**	**348**	**380**	**961**	**56.90%**	*high*	**low**
P34	13	563	206	*high*	*high*	P34	177	279	207	*high*	*high*	P34	196	199	545	57.98%	*high*	*high*
**P35**	**36**	**369**	**1**	*high*	**low**	**P35**	**3**	**338**	**92**	*high*	**low**	P35	9	19	380	93.14%	*high*	*high*
P36	0	38	1603	*high*	*high*	P36	1	948	1221	*high*	*high*	P36	942	654	924	36.67%	low	low
P37	7	2086	64	*high*	*high*	P37	32	1579	578	*high*	*high*	P37	201	228	2417	84.93%	*high*	*high*
P38	20	492	75	*high*	*high*	P38	27	416	162	*high*	*high*	P38	488	1019	27	1.76%	low	low

Bim and Mcl-1 are grouped in the last two classes and P-ERK in the first two classes for comparison with expert estimation (last two columns in the table). Gray labels indicate discordant cases between expert and IP analysis.

The P-ERK table contains a supplementary column entitled “R 50%” because the labeled “high” was validated only if the main class contained 50% of the hexagon patterns. Four, eight, and seven cases were found to be discordant for the WSI stained by the DAB relative to Bim, Mcl-1, and P-ERK respectively (**Table 4**).

#### Second approach: use of immunostaining scores

**Figure 5:**
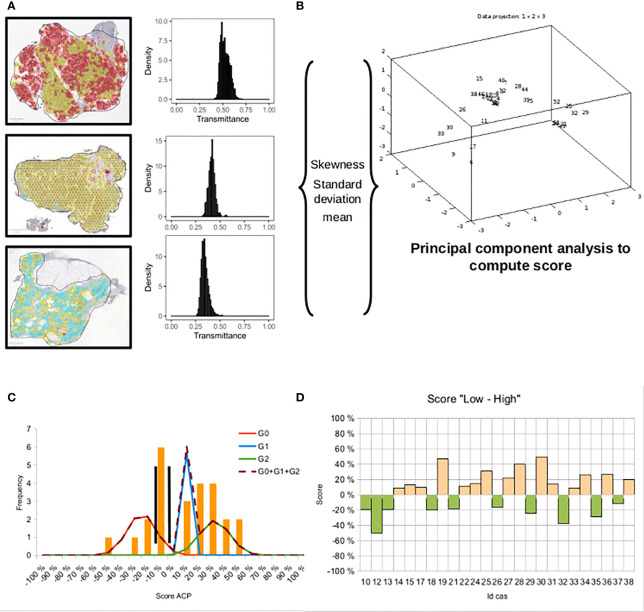
Complete procedure to compute an objective score taking distribution of staining of a specific marker in hexagons into account. **(A)** On each section, intensity of staining in hexagons was computed to obtain distribution of transmittances. **(B)** Shape parameters of distribution were extracted for each section, and a principal component analysis was used to compute a score. **(C)** Distribution of scores was obtained from component 1 of PCA. To classify the patients as low or high score, a mixture of Gaussians was applied to find thresholds. **(D)** The highest threshold was selected and used as the 0-value. Low scores are negative, and high scores are positive.

The second approach used the PCA algorithm (**Figure 5**). First, the first three statistical moments were computed (means, standard deviation, and skewness) from each histogram of the hexagon patterns, case per case. Second, the PCA algorithm was applied to the three statistical moments to find their best linear combination by exploiting the first principal factor. This procedure gave the biological score (**Table 5**).

**Table 5:** First three statistical moments in columns on left, values computed by main principal component on right and loadings (weight of statistical moments) for principal component in the last line.

Bim	I_Moy	I_Sig	I_Skw	CP1	Mcl-1	I_Moy	I_Sig	I_Skw	CP1	P-ERK	I_Moy	I_Sig	I_Skw	CP1
P10	0.37	0.035	0.178	-1.208	P10	0.316	0.026	1.307	-2.74	P10	0.009	0.055	6.372	-4.08
P12	0.312	0.025	0.679	-2.795	P12	0.36	0.035	1.193	-1.47	P12	0.185	0.188	0.083	-0.951
P13	0.335	0.04	0.208	-1.187	P13	0.402	0.037	0.738	-0.25	P13	0.356	0.227	-0.711	0.228
P14	0.476	0.044	0.129	0.263	P14	0.449	0.041	0.483	0.928	P14	0.269	0.172	-0.798	-0.23
P15	0.531	0.039	0.097	0.479	P15	0.411	0.034	1.235	-0.989	P15	0.423	0.146	-2.325	1.064
P17	0.435	0.058	0.642	0.296	P17	0.404	0.064	0.434	1.92	P17	0.449	0.069	-3.731	1.623
P18	0.414	0.033	0.488	-1.237	P18	0.47	0.04	1.467	-0.216	P18	0.344	0.205	-1.021	0.255
P19	0.561	0.055	-0.447	2.22	P19	0.471	0.048	0.481	1.648	P19	0.477	0.168	-2.162	1.301
P21	0.42	0.037	0.666	-1.148	P21	0.406	0.034	0.81	-0.498	P21	0.033	0.121	3.374	-2.894
P22	0.515	0.04	0.103	0.384	P22	0.465	0.04	0.923	0.449	P22	0.39	0.03	-0.278	0.123
P24	0.525	0.048	0.501	0.54	P24	0.384	0.039	0.626	-0.217	P24	0.267	0.25	-0.083	-0.429
P25	0.508	0.054	-0.155	1.433	P25	0.494	0.047	-0.13	2.683	P25	0.188	0.234	0.505	-1.051
P26	0.425	0.036	0.594	-1.065	P26	0.45	0.036	0.616	0.456	P26	0.131	0.172	0.606	-1.419
P27	0.449	0.053	-0.204	0.924	P27	0.436	0.03	0.97	-0.553	P27	0.235	0.21	-0.184	-0.588
P28	0.556	0.039	-1.087	1.844	P28	0.512	0.038	0.425	1.559	P28	0.46	0.169	-2.072	1.184
P29	0.425	0.042	1.374	-1.483	P29	0.363	0.036	1.405	-1.653	P29	0.268	0.178	-0.763	-0.241
P30	0.588	0.064	0.18	2.375	P30	0.425	0.035	-0.033	0.927	P30	0.432	0.176	-1.596	0.886
P31	0.452	0.042	-0.461	0.535	P31	0.397	0.033	1.297	-1.271	P31	0.398	0.169	-1.781	0.769
P32	0.344	0.036	0.988	-2.157	P32	0.361	0.033	0.865	-1.18	P32	0.298	0.161	-1.217	0.052
P33	0.494	0.045	0.391	0.249	P33	0.405	0.051	1.153	0.128	P33	0.39	0.205	-1.225	0.56
P34	0.464	0.056	-0.157	1.167	P34	0.41	0.058	0.185	1.948	P34	0.407	0.219	-1.106	0.617
P35	0.379	0.029	0.41	-1.68	P35	0.426	0.032	1.251	-0.936	P35	0.531	0.093	-3.931	2.13
P36	0.588	0.046	0.236	1.191	P36	0.452	0.042	1.242	0.049	P36	0.296	0.231	-0.444	-0.173
P37	0.426	0.036	0.303	-0.78	P37	0.427	0.034	1.364	-0.96	P37	0.469	0.133	-2.982	1.513
P38	0.445	0.05	-0.319	0.841	P38	0.422	0.031	0.294	0.238	P38	0.269	0.184	-0.729	-0.248
**CP**	**I_Moy**	**I_Sig**	**I_Skw**	** **	**CP**	**I_Moy**	**I_Sig**	**I_Skw**		**CP**	**I_Moy**	**I_Sig**	**I_Skw**	
1	63.68%	57.65%	-51.20%		1	-54.58%	-56.24%	62.11%		1	70.51%	3.84%	-70.81%	

**Figure 6:**
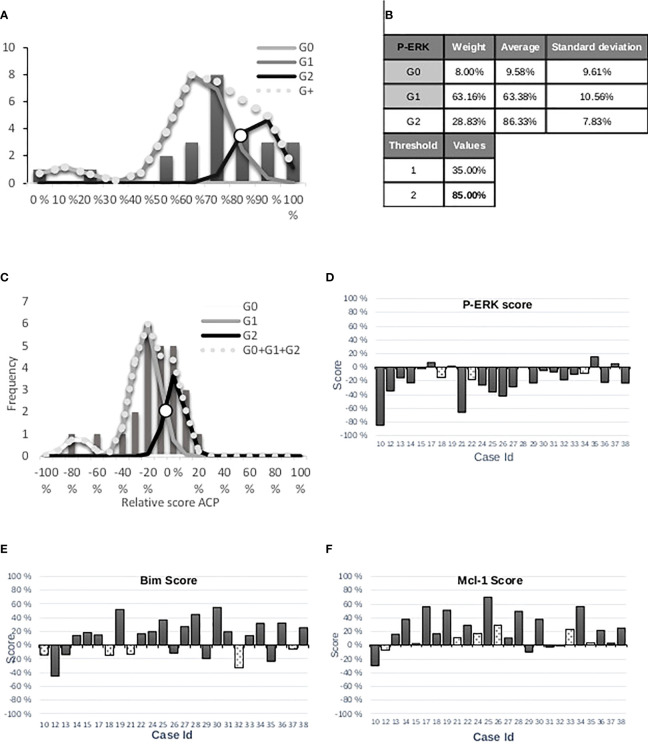
**(A)** Distribution of values of principal component 1 for P-ERK and three Gaussian functions. Automatic thresholds are determined with the algorithm of the Gaussian Mixture Model. **(B)** The threshold values correspond to crossing between Gaussians. **(C)** The highest threshold was selected and used as the 0-value. **(D)** Histogram representation of P-ERK marker score per patient by rapid reading. White bars corresponding to discordance between experts and IP. **(E)** Histogram representation of Bim marker score per patient. **(F)** Histogram representation of P-Mcl-1 marker score per patient.

The PCA values were normalized as a percentage to use the GMM. Three Gaussian functions and two thresholds were sought, as in the first approach (**Figure 6**).

Two automatic thresholds were adjusted in the neighborhood of the Gaussian curve intersection for the purpose of comparison with the estimation of the expert (identical to the first approach). Thus, each threshold delimited two areas: “low and high” tumor response for Bim (45%), Mcl-1 (25%), and P-ERK (85%), respectively. These thresholds were considered as the new origins to better assess the level relative to the tumor response, i.e., negative score for “low” and positive score for “high” (**Figure 6**). Five, six, and three cases were found discordant for the WSI stained by DAB relative to Bim, Mcl-1, and P-ERK, respectively (**Figure 6**).

## Discussion

We sought to develop a novel fully automated robust method for quantifying protein biomarker expression, here applied to the epithelial component of high-grade serous ovarian carcinomas (HGSOC). Our approach was based only on a cohort of 25 cases of HGSOC for which three biomarkers predictive of the response observed *ex vivo* to the BH3 mimetic molecule ABT-737 had been validated by a pathologist (12). This is a strength, since we tried to remain as close as possible to the results obtained by the pathologist. It is also a limitation, since variations are known to occur between two interpretations of a unique histological slice by the same pathologist and by two or more pathologists (7–9).

We calibrated our algorithms using stereology performed by two experts to detect both immunohistochemical staining and epithelial/stromal compartments, as recommended as a quality check (24). Analyses of this calibration processes showed a good correlation between the experts for both epithelium and immunostaining detection. Furthermore, there was a good correlation between the experts and IP, after excluding discordant results between them. This calibration process was essential, since it allowed the efficient detection of both epithelium and immunostained areas. Immunostaining was then quantified by using transmittance computing within hexagon grids. The GMM was then used to reproducibly establish low, medium, or high thresholds within each hexagon.

By comparing the classification obtained with these thresholds to the classification established by the pathologist, we observed four, eight, and seven discordant results in 25 cases for Bim, Mcl-1, and P-ERK, respectively.

With the GMM, we used histograms pooling all the hexagons from all the cases. Interestingly, the values of most of the hexagons in discordant cases were close to the thresholds, explaining why the class could be easily construed as discordant in these cases with the one defined by the pathologist. IP could thus allow variations in interpretation due to subjectivity to be avoided.

Other parameters could also explain these discrepancies. For instance, Mcl-1 expression is difficult to appreciate, particularly because its localization can vary from one case to another. Mcl-1 can be expressed in the cytosol, in the nucleus, or both, as observed in our study and by other groups (25). Whereas IP considers both types of staining, pathologists mainly evaluate cytosolic staining, so this could generate discrepancies.

For P-ERK immunostaining, the situation is more complex. The activation of P-ERK is often strongly correlated to survival signals transmitted by contact between cancer cells and the extracellular matrix and stromal components. While ERK phosphorylation is most frequently observed only in cancer cells, it may also be observed in stromal cells or in both. Moreover, its expression intensity can strongly vary from one area of the tumor to another. Pathologists must thus consider these features and the proportion of cancer cells highly expressing P-ERK when proposing a composite score (a “high” case will be a tumor sample in which more than 50% of cancer cells show a high level of P-ERK). This relatively subjective appreciation could of course be another source of discrepancy between IP and pathologists’ observations.

Another potential source of discordance is that, in the classification of immunostainings as low, medium, or high, there is no appreciation of an eventual intra-tumor heterogeneity, yet this could be decisive in the therapeutic management of patients (26, 27). For example, a patient presenting a low expression of Mcl-1 in 100% of their cancer cells could be globally more sensitive to ABT-737 than another in whom 30% of cancer cells strongly express Mcl-1. Both cases would be classified as “low” by the pathologist. In this setting, IP could easily decipher the respective proportions of low and high areas and include this parameter in a composite score. Therefore, we included three statistical moments (case per case) in the process: mean value, standard deviation, and skewness parameters of the distribution values of transmittance within each hexagon. By doing so, we were able to use PCA to find the best combination of the three parameters. The first factor component was therefore relevant to establish a score linked to each staining. To fit with the pathologist scores, the GMM was also applied to establish thresholds. This approach allowed us to decrease the levels of discrepancy for both Mcl-1 and P-ERK, i.e., taking heterogeneity into account increased the agreement with the pathologist.

In conclusion, this objective, simple, robust, and calibrated method using simple tools and known parameters can be used to quantify and characterize the expression of protein biomarkers within different tumor compartments, using a mathematical definition of thresholds and taking into account the intra-tumoral heterogeneity of staining. It is replicable and could be used in other biological or medical settings after further validation. It is non-subjective, uses a quality control of proven interest, has a fully automated choice of thresholds, and has defined composite scores, thus allowing the intra-tumor heterogeneity of immunostaining to be taken into account. This fully automated approach could help to avoid the misclassification of patients and the subsequent negative impact on their therapeutic care. A development for the future would be to analyze the PCA score by deep learning in connection with medical data to help pathologists establish the right diagnosis.

## Data availability statement

The original contributions presented in the study are included in the article/supplementary material. Further inquiries can be directed to the corresponding authors.

## Ethics statement

The studies involving human participants were reviewed and approved by Consent number: NCT01440504. The patients/participants provided their written informed consent to participate in this study.

## Author contributions

NE: Image processing and written of the manuscript. FG: Immunostaining of virtual slides (WSI) and written of the manuscript. CB-F: Senior pathologist. P-MM: Written of the manuscript. P-EB: Stereology and staining evaluation. SD: Immunostaining of virtual slides (WSI). BP: Algorithm development and written of the manuscript. LP: Stereology, staining evaluation and written of the manuscript. All authors contributed to the article and approved the submitted version.

## Funding

P-MM was supported by the Cancer Institut Thématique Multi-Organisme of the French National Alliance for Life and Health Sciences (AVIESAN) Plan Cancer 2014-2019 (doctoral grant). This work is part of the “ONCOTHERA” European project, co-funded by the Normandy County Council, the European Union within the framework of the Operational Program ERDF/ESF 2014-2020. It is also funded by Ligue Contre le Cancer, Calvados Committee.

## Conflict of interest

The authors declare that the research was conducted in the absence of any commercial or financial relationships that could be construed as a potential conflict of interest.

The handling editor AL declared a past co-authorship with the author BP.

## Publisher’s note

All claims expressed in this article are solely those of the authors and do not necessarily represent those of their affiliated organizations, or those of the publisher, the editors and the reviewers. Any product that may be evaluated in this article, or claim that may be made by its manufacturer, is not guaranteed or endorsed by the publisher.
